# Attenuation of Doxorubicin-Induced Cardiotoxicity by mdivi-1: A Mitochondrial Division/Mitophagy Inhibitor 

**DOI:** 10.1371/journal.pone.0077713

**Published:** 2013-10-17

**Authors:** Mayel Gharanei, Afthab Hussain, Omar Janneh, Helen Maddock

**Affiliations:** 1 Department of Biomolecular and Sport Sciences, Coventry University, Coventry, United Kingdom; 2 Division of Biomedical Sciences, St. George's, University of London, London, United Kingdom; National Institutes of Health, United States of America

## Abstract

Doxorubicin is one of the most effective anti-cancer agents. However, its use is associated with adverse cardiac effects, including cardiomyopathy and progressive heart failure. Given the multiple beneficial effects of the mitochondrial division inhibitor (mdivi-1) in a variety of pathological conditions including heart failure and ischaemia and reperfusion injury, we investigated the effects of mdivi-1 on doxorubicin-induced cardiac dysfunction in naïve and stressed conditions using Langendorff perfused heart models and a model of oxidative stress was used to assess the effects of drug treatments on the mitochondrial depolarisation and hypercontracture of cardiac myocytes. Western blot analysis was used to measure the levels of p-Akt and p-Erk 1/2 and flow cytometry analysis was used to measure the levels p-Drp1 and p-p53 upon drug treatment. The HL60 leukaemia cell line was used to evaluate the effects of pharmacological inhibition of mitochondrial division on the cytotoxicity of doxorubicin in a cancer cell line. Doxorubicin caused a significant impairment of cardiac function and increased the infarct size to risk ratio in both naïve conditions and during ischaemia/reperfusion injury. Interestingly, co-treatment of doxorubicin with mdivi-1 attenuated these detrimental effects of doxorubicin. Doxorubicin also caused a reduction in the time taken to depolarisation and hypercontracture of cardiac myocytes, which were reversed with mdivi-1. Finally, doxorubicin caused a significant elevation in the levels of signalling proteins p-Akt, p-Erk 1/2, p-Drp1 and p-p53. Co-incubation of mdivi-1 with doxorubicin did not reduce the cytotoxicity of doxorubicin against HL-60 cells. These data suggest that the inhibition of mitochondrial fission protects the heart against doxorubicin-induced cardiac injury and identify mitochondrial fission as a new therapeutic target in ameliorating doxorubicin-induced cardiotoxicity without affecting its anti-cancer properties.

## Introduction

The anthracycline antibiotic doxorubicin is used to treat a wide variety of cancers, but reports of its cardiotoxic properties compromises its clinical utility [1–3]. The cardiotoxic effects of doxorubicin are thought to be mediated via disruption of the mitochondrial function [4]. Previous studies have also shown doxorubicin to cause cardiotoxicity through the generation of free radicals [5], stimulation of lipid peroxidation [6] and alteration and disruption of cellular membrane integrity [7]. Arrhythmias, hypotension and depression of the contractile function are some of the acute effects of doxorubicin-induced cardiotoxicity [8], while chronic heart failure and dilative cardiomyopathy are more common and severe in patients who are on long term anthracyclines treatment [9,10]. Large scale clinical trials have shown that doxorubicin induced cardiotoxicity is irreversible and dose dependent [11]. 

Due to advances in basic and clinical cancer research, cancer and malignancies are becoming more manageable, unfortunately the adverse cardiovascular effects of systemic anticancer agents are still a serious concern [12,13]. Thus it is imperative to understand the cellular and molecular basis of doxorubicin-induced cardiotoxicity with the view to finding therapies that would offer cardioprotection without affecting its anti-tumour effects. Interventions using β blockers, free radical scavengers, antioxidants and renin-angiotensin system inhibitors have met with limited success due, not only, to side effects but also because of their negative interactions with doxorubicin [14]. While aiming to reduce the cardiotoxic effects of anthracyclines using adjunct therapies, it is imperative to assess the effects in cancer cell line to ascertain the clinical utility of such treatments. Interestingly, recent studies using the phosphodiesterase-5 inhibitors sildenafil or tadalafil have shown promise by showing a reduction in the cardiotoxic effects of doxorubicin without affecting its anti-cancer activity [15,16]. 

Cell death pathways activated by doxorubicin treatment usually involve the mitochondria to initiate apoptosis or necrosis [17,18]. Mitochondrial dynamics are found to play an essential role in cellular function and apoptosis [19]. In order to maintain mitochondrial integrity and efficiency, a constant interplay between mitochondrial fission and fusion is important. Previous studies have demonstrated that upon induction of oxidative stress or ischaemia, dynamin related protein 1 (Drp1), a protein involved in mitochondrial fission, translocates from the cytosol to the mitochondria to execute the mitochondrial division process [20]. This involves hydrolysing GTP [21–23], which dysregulates the balance between mitochondrial fusion and fission. Mitochondrial fission leads to cytochrome c release and activation of caspases, which can ultimately lead to cell death [20,24,25]. Studies also reported that the dominant negative form of Drp1, Drp_K38A_, had the ability to inhibit mitochondrial division [20] suggesting a regulatory role for Drp1 in mitochondria-mediated apoptosis. Additionally, inhibition of mitochondrial division either genetically or pharmacologically with the mitochondrial division inhibitor 1 (mdivi-1) has been found to inhibit cell death in models of ceramide-induced toxicity [17] and myocardial ischaemia and reperfusion injury [26]. Furthermore, it was recently shown that treatment with mdivi-1 protected against pressure induced heart failure by ameliorating left ventricular dysfunction and promoting angiogenesis [27]. Increase in apoptosis and abnormal mitophagy noticed in pressure overload samples were also prevented when treated with mdivi-1 [27].

Cancers are likely to develop in the later stages of life, when the chances of developing heart diseases are equally high [28]. Patients with pre-existing heart diseases are usually excluded or underrepresented in clinical trials, which aim to identify the efficacy and potential adverse effects of drugs. We have recently shown that doxorubicin administration at reperfusion exacerbates ischaemia reperfusion injury, which was prevented when co-administered with cyclosporin A [29]. It is therefore necessary to investigate the off-target effects of anti-cancer therapeutics or adjunct therapies in stressed or diseased conditions such as ischaemia and reperfusion injury.

Given that doxorubicin-induced cardiotoxicity may be mediated by an imbalance in mitochondrial fusion and fission, we investigated the effects mdivi-1 on doxorubicin-induced cardiotoxicity using the Langendorff model in naïve and in conditions of ischaemia and reperfusion injury. A model of oxidative stress was used to record the time taken to depolarisation and hypercontracture of cardiac myocytes upon drug treatment and western blot analysis was used to evaluate the levels signalling proteins. Data on the effects of mdivi-1 on the cytotoxicity of doxorubicin was also assessed in HL60 cell line.

## Methods

### Chemicals

Doxorubicin hydrochloride and mdivi-1 were purchased from Tocris Cookson (Bristol, UK). Mdivi-1 was dissolved in DMSO, ensuring that the final concentration of DMSO was less than 0.01% during the experiments. Doxorubicin was dissolved in ultra-pure water. The dissolved drugs were aliquotted and stored at -20°C. All other reagents unless otherwise stated were obtained from Fisher Scientific (UK). All western blot reagents were purchased from Bio-Rad, UK. Antibodies were from New England Biolabs, UK.

### Animals

Male Sprague-Dawley rats (350-400g body weight) were used in all experiments. Experiments were conducted in accordance with the guidelines on the operation of animals (Scientific Procedures Act 1986) and approved by the Coventry University biological resource animal welfare and ethics committee. Animals were obtained from Charles River UK Limited (Margate, UK) and kept in standard laboratory cages at Coventry University.

### Isolated perfused heart preparation

Rats were sacrificed by schedule one method of cervical dislocation and the hearts were rapidly excised and placed into ice-cold Krebs Henseleit (KH) buffer. The hearts were quickly mounted onto the Langendorff system and retrogradely perfused with KH buffer (118.5mM NaCl, 25mM NaHCO_3_, 4.8mM KCl, 1.2mM MgSO_4_, 1.2mM KH_2_PO_4_, 1.7mM CaCl_2_, and 12mM glucose, pH 7.4). KH buffer was gassed using 95% O_2_ and 5% CO_2_ and maintained at 37°C at all times. The left atrium was trimmed away and a latex iso-volumic balloon was carefully introduced into the left ventricle and inflated up to 5-10mmHg. Functional recordings were taken via a physiological pressure transducer connected to Powerlab (AD Instruments Ltd. Chalgrove, UK). Left ventricular pressure, heart rate and coronary flow were measured at regular intervals. The hearts used for the naïve experiments were weighed and frozen at the end of the experimental protocol.

To simulate ischaemia and reperfusion protocol a snare was used to tighten to left descending coronary artery to induce ischaemia and release of the snare initiated reperfusion. At the end of reperfusion ± drug treatment the snare was tightened to re-occlude the coronary artery branch. A 1ml solution of 0.25% Evans blue was then infused slowly via the aorta to delineate the non-risk zone of the myocardium, which stained dark blue.

The hearts were weighed and frozen at -20°C until used. The frozen hearts were then sliced into 2mm thick transverse sections and incubated in triphenyltetrazolium chloride (TTC) solution (1% in phosphate buffer) at 37°C for 10 - 12 min and fixed in 10% formalin for at least 4 hours. The tissue at risk stained red and infarct tissue appeared pale. The risk zone and infarct areas were traced onto acetate sheets. Using computerised planimetry (Summasketch II, Summagraphics), the percentage of infarct to risk ratio (%) was calculated. 

### Langendorff protocol

**Figure 1:**
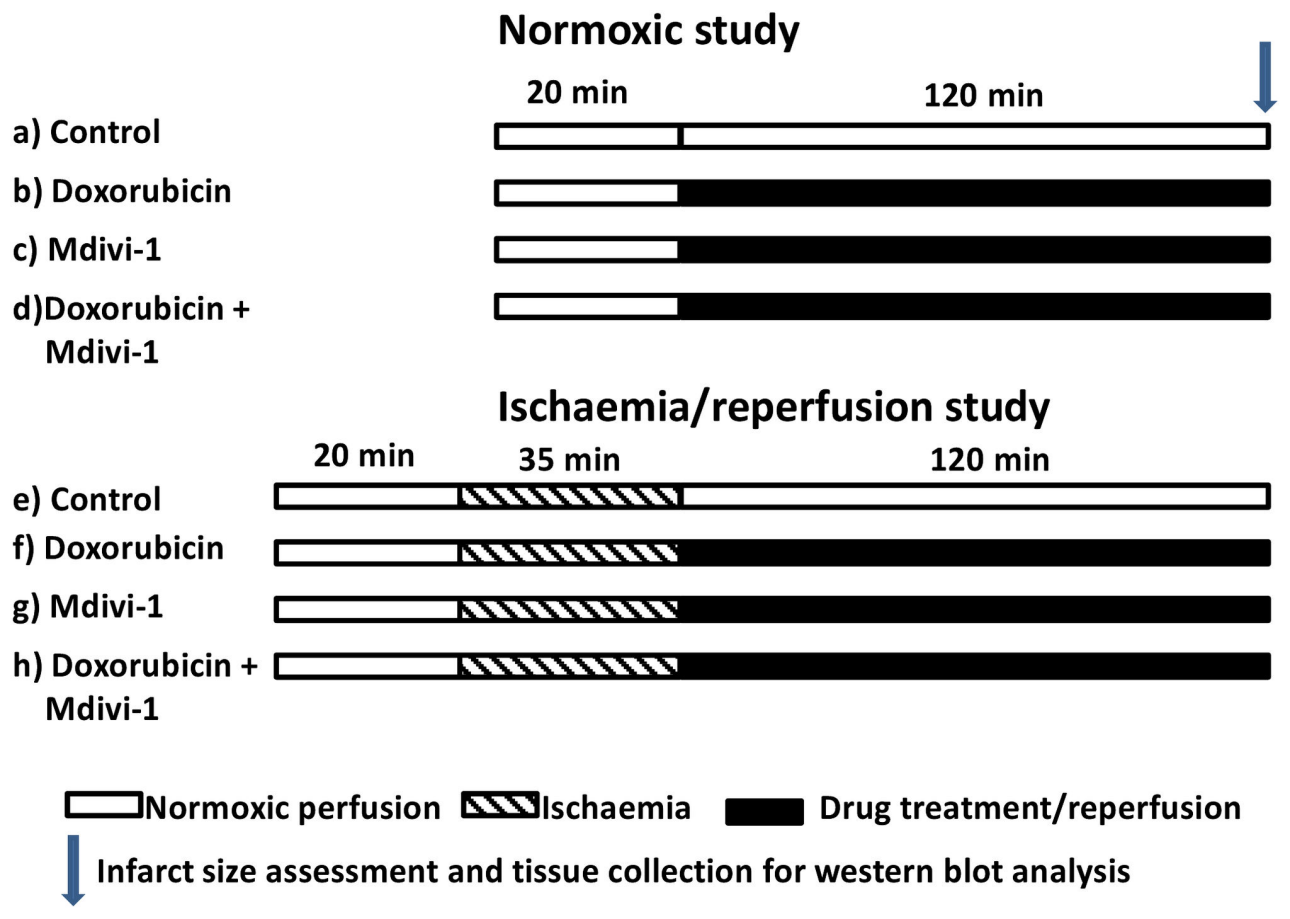
Treatment protocol for the naïve and ischaemia/reperfusion Langendorff studies. Naïve: 20 minutes of stabilisation followed by 120 minutes of drug treatments or treatment with vehicle control. Ischaemia/reperfusion: 20 minutes stabilisation, 35 minutes ischaemia and 120 minutes of reperfusion ± drug treatments.

All hearts were allowed to stabilise for 20 minutes prior to being subjected to vehicle or drug treatment for 120 minutes (normoxic protocol) or 35 minutes regional ischaemia followed by 120 minutes reperfusion ± drug treatment (IR protocol). The hearts were randomly assigned to the following groups; 1) normoxic protocol: a) hearts perfused with KH buffer (control) for 140 minutes; b) hearts perfused with doxorubicin (1µM) for 120 minutes following 20 minutes of stabilisation; c) hearts perfused with mdivi-1 (1µM) for 120 minutes following 20 minutes of stabilisation and, d) hearts perfused with doxorubicin (1µM) in presence of mdivi-1 (1µM) for 120 minutes following the stabilisation period. 2) Or hearts underwent an ischaemia/reperfusion protocol: e) Perfusion with KH buffer alone for 120 minutes following 20 minutes of stabilisation and 35 minutes of regional ischaemia; f) hearts perfused with doxorubicin (1µM) throughout the duration of reperfusion; g) hearts perfused with mdivi-1 (1µM) throughout the duration of reperfusion and, h) hearts perfused with doxorubicin (1µM) in presence of mdivi-1 (1µM) throughout the duration of reperfusion (Figure 1).

### Isolation of adult rat cardiac myocytes

Adult rat cardiac myocytes were isolated by enzymatic digestion, as described previously [30]. Briefly, male Sprague Dawley rats (350-400g body weight) were sacrificed by cervical dislocation and the hearts were quickly excised and mounted on a modified Langendorff apparatus. Then the hearts were perfused with modified KH bicarbonate buffer (116mM NaCl, 25.0mM NaHCO_3_, 5.4mM KCl, 0.4mM MgSO_4_ .7.H_2_O, 1.7mM CaCl_2_, 10mM glucose, 20mM taurine, 5mM pyruvate and 0.9mM Na_2_HPO_4_.12H_2_O, pH 7.4). The buffer was oxygenated with 95% O_2_ and 5% CO_2_; 37°C. Hearts were perfused for 5 minutes with calcium free KH buffer, upon perfusion with the calcium free buffer the heart ceased contraction. The hearts underwent a final 5 minute perfusion cycle with modified KH digestion buffer (0.075% Worthington’s Type II Collagenase, 4.4μM CaCl_2_, pH 7.4). During perfusion with collagenase the effluent was collected and reused. 

After perfusion with collagenase the heart was removed and the atria were cut away. The ventricles were teased apart and incubated with fresh KH digestion buffer for 10 minutes on an orbital shaker. The digestion buffer was aspirated and passed through a nylon mesh and the filtrate was collected and centrifuged at 400 rpm for 2 minutes. The supernatants were removed using a sterile pipette and the pellet was redistributed in 15ml freshly prepared restoration buffer (116mM NaCl, 25.0mM NaHCO_3_, 5.4mM KCl, 0.4mM MgSO_4_ .7.H_2_O, 10mM glucose, 20mM taurine, 5mM pyruvate 0.9mM Na_2_HPO_4_.12H_2_O, 1% BSA and 1% Pen-Strep, pH 7.4). The calcium concentration was gradually brought to 1.25 mM to avoid calcium overload. The isolated myocytes were incubated at 37 °C, 5% CO_2_, for 24 hours before being used for protocol to assess depolarisation and hypercontraction of cardiac myocytes. 

### Protocol for oxidative stress induced depolarisation and hypercontraction

Oxidative stress in response to the positively charged fluorescent dye, tetramethylrhodamine methyl ester (TMRM) and laser illumination was used to record the time taken to depolarisation and hypercontraction of cardiac myocytes [31]. Briefly, TMRM was used as it penetrates and concentrates in negatively charged mitochondria due to its charged nature. Laser illumination causes the TMRM to release ROS from the mitochondria, leading to depolarisation of the mitochondrial membrane. The release of TMRM along with the content of the mitochondria into the cytoplasm can be observed as an increase in fluorescence intensity on the confocal microscope. Oxidative stress was continued until the cells underwent hypercontracture, marking the point of ATP depletion and cell death. The time taken to depolarisation and hypercontracture were recorded. 

Following the overnight incubation of the isolated cardiac myocytes, the cells were transferred to laminin-coated cover slips and allowed to adhere for 3 hours prior to being prepared for drug treatment and microscopy. The adherent cardiac myocytes were then incubated for 15 minutes in microscopy buffer (KH buffer supplemented with 10 mM HEPES and 1.2µM CaCl_2_, pH 7.4) containing 3µM TMRM. The TMRM was then washed away and the cells were incubated without or with the drugs for 10 minutes before being placed on the confocal microscope.

Cells were assigned to the following groups: Control group, incubated with microscopy buffer alone for 10 minutes; incubation with 1µM doxorubicin and in presence of mdivi-1 (1µM) or incubation with mdivi-1 (1µM) alone. 

### Confocal microscope

The cover-slips including the adherent myocytes were placed on the heated stage (37°C) on a Zeiss 510 CLSM confocal microscope equipped with 20x objective lens (NA 1.3). 543-nm emission line of HeNe laser was used to initiate oxidative stress. A 585-nm long pass filter was used to collect TMRM fluorescence. All confocal settings were kept constant throughout the experiments to ensure comparability. Images were analysed using Zeiss software (LSM 2.8)

### Assessment of HL60 cells viability

HL60 cells (10,000 cells/well) were incubated in the presence and absence of the drugs (alone and in combination) in a 96 well microtitre plate for 24h. After 24h, 20µl of 5mg/ml MTT was added to each well and incubated for 2h. Thereafter, 100µl of lysis buffer (containing 15% SDS in 50% dimethyl formamide) was added to each well and incubated overnight before the absorbance was read at 492nm. Data was analysed and the background absorbance was subtracted from the values obtained, which were then normalised against control.

### Western blot analysis

Approximately 50 mg of the frozen heart sample was used for protein extraction by homogenizing the samples in suspension buffer (100mM NaCl, 10mM TRIS, 1mM ethylenediaminetetraacetic acid, (pH 8.0), 2mM Sodium Pyrophosphate, 2mM Sodium Fluoride, 2mM ß-Glycerophosphate and protease inhibitor cocktail followed by high speed centrifugation. The supernatants were assessed for protein content using nanoDrop spectrophotometer ND1000 (Thermo Scientific). Then 60µg of the protein was loaded into the wells of the precast gels 4-12% (Bio-Rad, UK) and separated at 130mV for 90minutes. After separation, the proteins were transferred to The Hybond-P Polyvinyl Difluoride membrane (PVDF) using Trans-Blot Turbo Transfer system (Bio-Rad) and probed for the phosphorylated and total form of Akt (Ser_473_) and Erk 1/2 (Thr_202_/Thr_204_). The relative changes in the phosphorylated Akt and Erk 1/2 protein levels were measured and corrected for differences in protein loading as established by probing for total Akt and Erk 1/2 respectively.

### Flow cytometric analysis

Following the isolation of adult ventricular myocytes, the cells were incubated in the absence or the presence of doxorubicin (1µM), mdivi-1 (1µM) and the combination of doxorubicin and mdivi-1 in an atmosphere of 37 °C, 21 % O_2_ and 5 % CO_2_ for 4 hours. Following the drug treatment period, the cells were harvested into labelled eppendorff tubes and centrifuged at 1200 rpm for 2 minutes. The cells were then resuspended in phosphate buffered saline (PBS) and fixed with 3% formaldehyde for 10 minutes at room temperature. Finally, the cells were permeabilised by incubation with ice-cold 90% methanol for 30 minutes at 4 °C followed by freezing the samples at -20 °C to be analysed the following day.

The following day, the cells were washed 2x with incubation buffer (0.5 % BSA in PBS) followed by blocking with the incubation buffer at room temperature for 10 minutes. The cells were then probed with the primary anti-body (phospho-Drp1 and phospho-p53) at 1:500 dilutions in incubation buffer at room temperature for 1 hour. The cells were washed 2x with the incubation buffer and resuspended in alexa flour@ conjugated secondary antibody at 1:1000 dilution for 30 minutes at room temperature. Following the final 2x washes with the incubation buffer, the cells were resuspended in PBS and analysed with the flow cytometer on the FL1 channel.

### Statistical analysis

The data were expressed as mean ± SEM. Infarct size, the times taken to depolarisation and hypercontracture and the western blot data were tested for group differences using one way analysis of variance (ANOVA) with Fishers post hoc tests. Left ventricular developed pressure, heart rate and coronary flow were assessed for statistical difference using one-way analysis of variance (ANOVA) for each time point. A p-value of p<0.05 was considered statistically significant. 

## Results

### Mdivi-1 protects against doxorubicin induced myocardial dysfunction and infarction in naïve conditions

**Figure 2:**
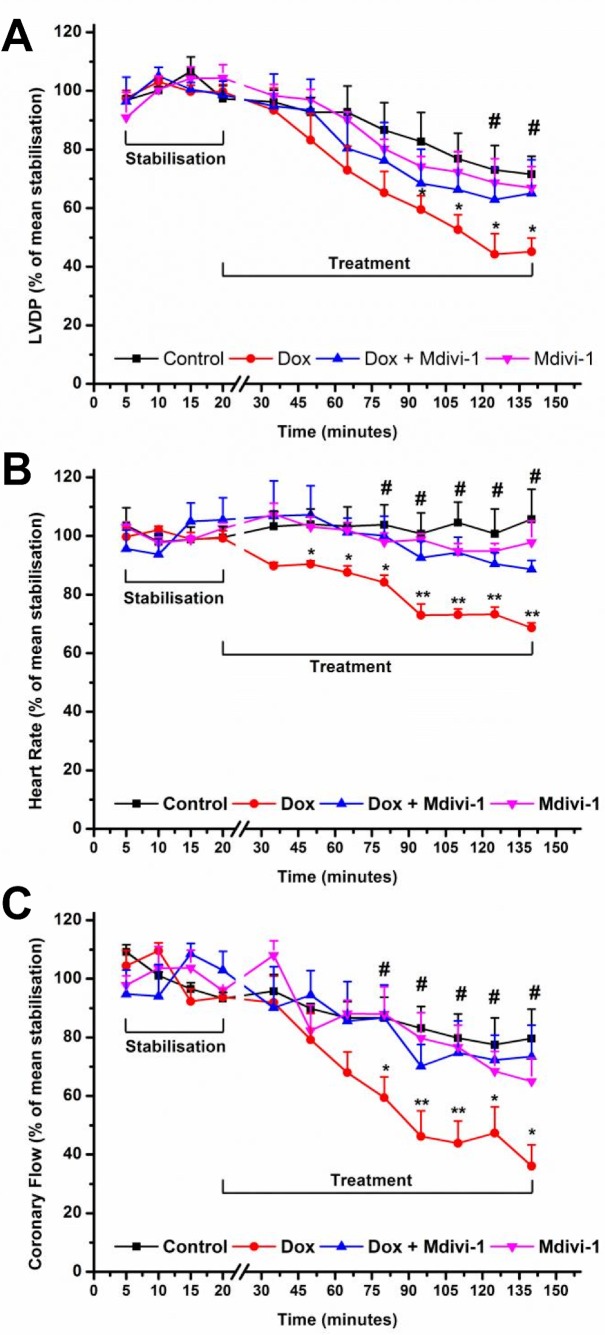
The effects of Doxorubicin (Dox), Mdivi-1 and the co-administration of Dox + Mdivi-1 on the haemodynamics of the heart. The hearts underwent 20 minutes of stabilization followed by 120 minutes perfusion in the presence absence of drugs (alone and in combination).The effects of drug treatment on left ventricular developed pressure, b) heart rate and c) coronary flow as a percentage of the mean stabilization period. *p<0.05 vs. time matched control, **<0.01 vs. time matched control, #p<0.05 vs. time matched doxorubicin. n= 6-8.

The effects of doxorubicin (alone and in combination with mdivi-1) on LVDP recording is demonstrated in Figure 2a. We observed that doxorubicin caused a significant (p<0.05 vs. time matched control) drop in LVDP after 90 minutes of drug treatment (at 120 minutes of treatment; 45.1 ± 4.7% vs. 71.6 ± 6.1%, respectively, p<0.05, Figure 2a) which was attenuated by co-administration with mdivi-1 (at 120 minutes; 45.1 ± 4.7% vs. 65.1 ± 11.4%, respectively, p<0.05, Figure 2a). Treatment with mdivi-1 alone did not appear to have a significant effect on LVDP as compared to control (66.9 ± 7 % vs. 71.6 ± 6.1%, respectively, Figure 2a). 

Doxorubicin treatment caused a significant reduction in the heart rate as compared to time matched controls (at 120 minutes; 68.6 ± 1.7% vs. 105.7 ± 10.3%, respectively, p<0.01; Figure 2b). Figure 2b also shows that co-administration of doxorubicin with mdivi-1 significantly reversed the effects of doxorubicin alone on heart rate (at 120 minutes; 88.6 ± 3% vs. 68.6 ± 1.7%, respectively, p<0.05, Figure 2b), while mdivi-1 alone did not have an effect on heart rate when compared to control (97.9 ± 7% vs. 105.7 ± 10.3%, respectively, Figure 2b). Doxorubicin treatment also caused a significant reduction (p<0.05) in the coronary flow after 75 minutes of treatment as compared to time matched control values (at 120 minutes; 36 ± 7.3% vs. 79.6 ± 10%, respectively, p<0.05, Figure 2c), which was attenuated when doxorubicin was co-administered with mdivi-1 (at 120 minutes; 36 ± 7.3% vs.73.9 ± 10.8%, respectively, p<0.05, Figure 2c), while mdivi-1 alone did not have an effect when compared to control (64.9 ± 8% vs. 79.6 ± 10%, respectively, Figure 2c) .

**Figure 3:**
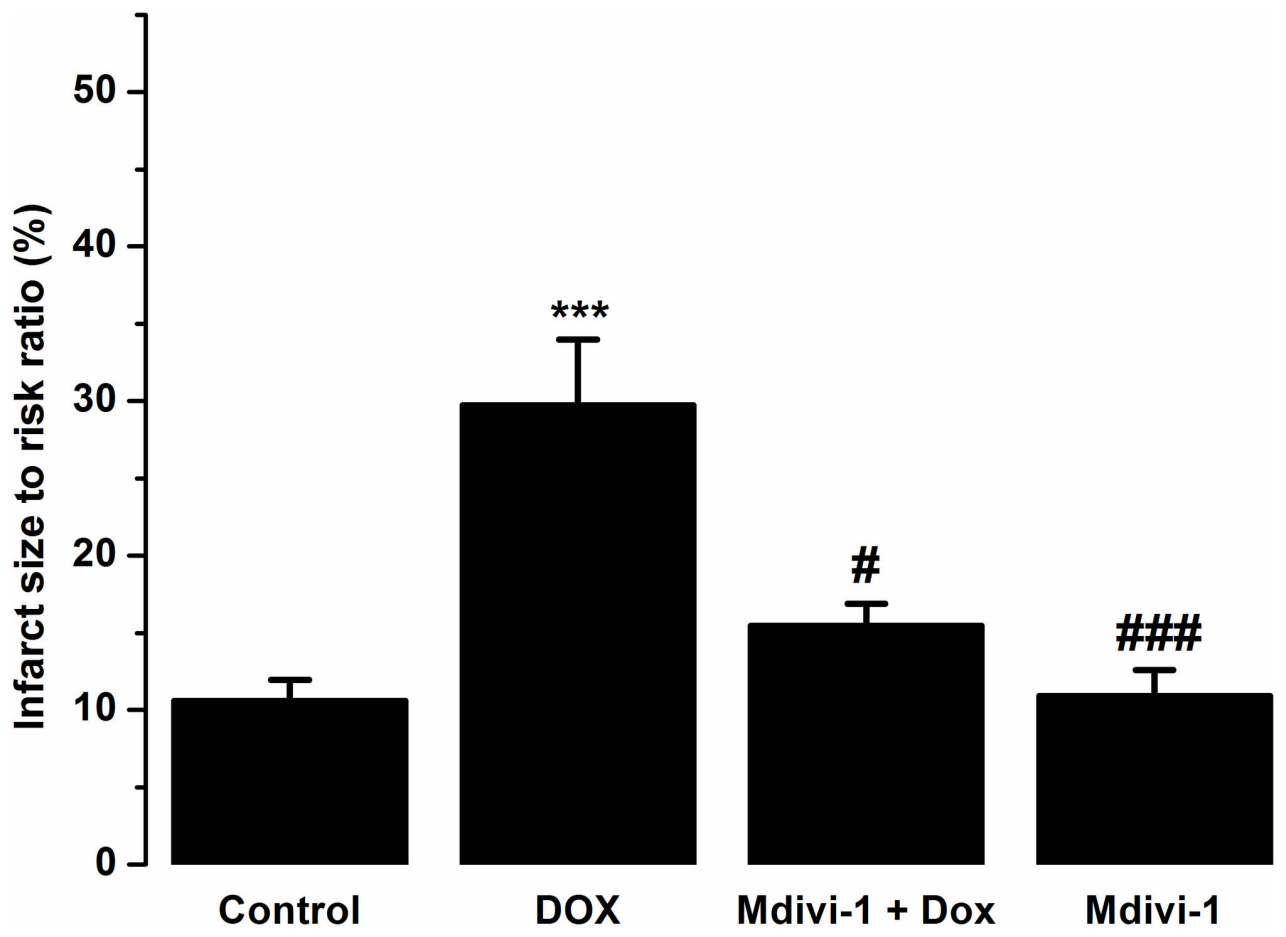
The effects of Dox, Mdivi-1 and the co-administration of Dox with Mdivi-1 on the infarct to risk ratios in the isolated heart Langendorff model. ***p<0.001 vs. control, #p<0.05 vs. doxorubicin, ###p<0.001 vs. doxorubicin n= 6-8.

Figure 3 shows the effects of doxorubicin (alone and in combination with mdivi-1) on infarct to risk ratio in the naïve Langendorff hearts. Doxorubicin (1µM) caused a significant (p<0.001) increase in the infarct size in the left ventricle as compared to control (29.74 ± 4.2% vs. 10.6 ± 1.34%, respectively, Figure 3) which was significantly (p<0.05) reversed (29.74 ±4.2% vs. 15.4 ±1.4 %, respectively, Figure 3) by mdivi-1 co-administration.

### Mdivi-1 protects against doxorubicin-induced cardiotoxicity in the conditions of ischaemia/reperfusion injury

**Figure 4:**
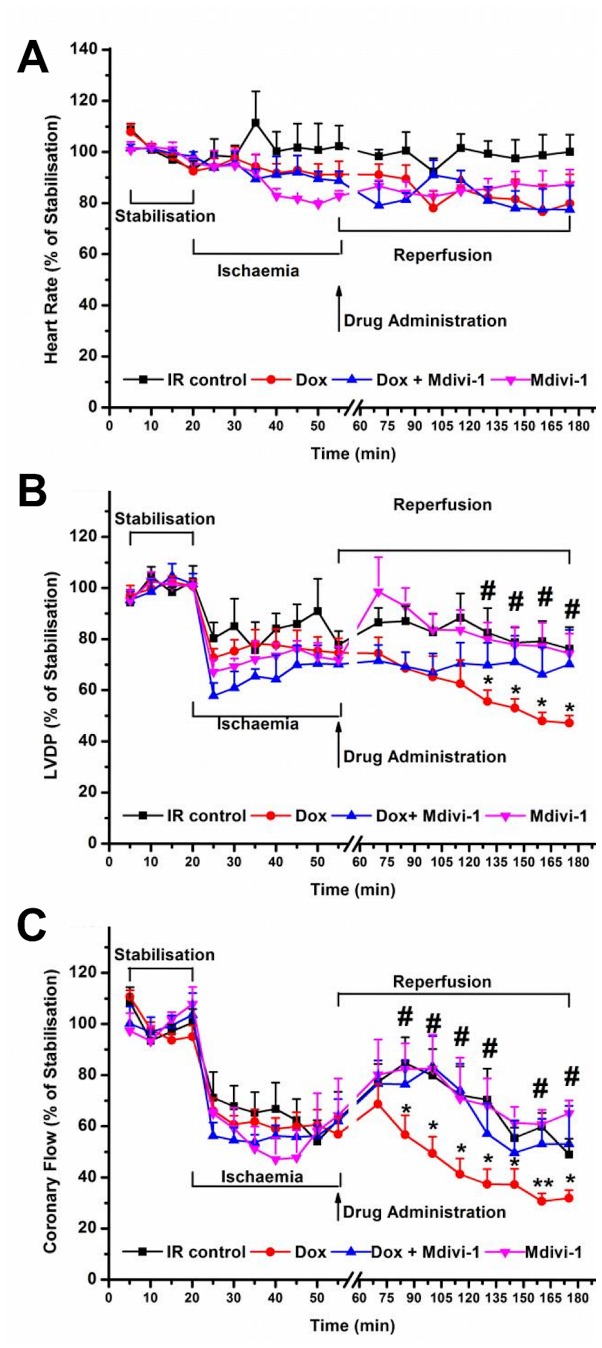
The effects of Doxorubicin (Dox), Mdivi-1 and the co-administration of Dox + Mdivi-1 on the haemodynamics of the heart during ischaemia and reperfusion. The hearts underwent 20 minutes of stabilization, 35 of ischaemia and 120 minutes of reperfusion, drugs are administered throughout reperfusion period. a) The effects of drug treatment on heart rate, b) left ventricular developed pressure (LVDP) and c) coronary flow as a percentage of the mean stabilization period. *p<0.05 vs. time matched IR control, **<0.01 vs. time matched IR control. n= 6-8. IR, ischaemia/reperfusion; Dox, doxorubicin.

**Figure 5:**
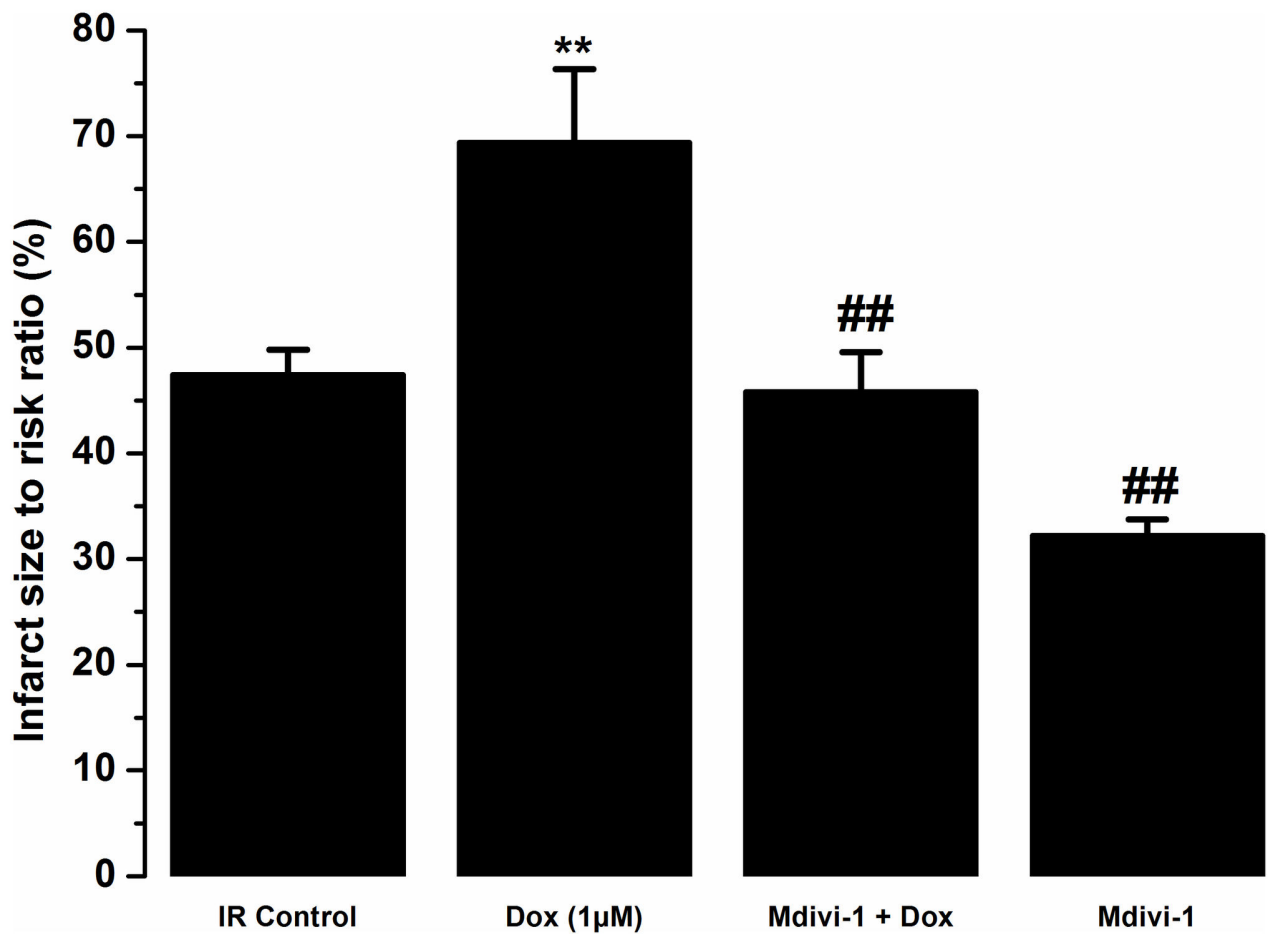
The effects of Dox, Mdivi-1 and the co-administration of Dox and Mdivi-1 on the infarct to risk ratios in the whole heart Langendorff model of ischaemia/reperfusion injury. **p<0.001 vs. IR control and ##p<0.01 vs. doxorubicin. n= 6-8. IR, ischaemia/reperfusion; Dox, doxorubicin.

Haemodynamic data recorded for tested agents in the presence of ischaemia/reperfusion injury is presented in Figure 4. We observed no significant difference for the heart rate values recorded from all the treatment groups (Figure 4a). Doxorubicin treatment during the reperfusion caused a significant reduction in the LVDP as compared to time-matched control from 45 minutes into reperfusion (at 120 minutes of treatment; 47.3 ± 3% vs. 76.3 ± 8.6%, respectively, p<0.05, Figure 4b). Co-treatment with mdivi-1 attenuated the reduction in LVDP observed with doxorubicin alone (at 120 minutes of treatment; 70.3 ± 13.5% vs. 47.3 ± 3%, respectively, p<0.05, Figure 4b). As observed in the naïve study, doxorubicin treatment also caused a significant reduction in the coronary flow following ischaemia/reperfusion injury as compared to time-matched controls (at 120 minutes; 32 ± 3.2% vs. 49.1 ± 6.2%, respectively, p<0.05), which was significantly reversed by co-treatment with mdivi-1 (at 120 minutes; 32 ± 3.2% vs. 53.3 ± 14. 8%, respectively, p<0.05, Figure 4c). Doxorubicin treatment during the reperfusion phase significantly increased (P<0.01) infarct size to risk ratio as compared to vehicle control (69.4 ± 7% vs. 47.4 ± 2.5%, respectively, Figure 5). Treatment with mdivi-1 alone during the reperfusion phase significantly reduced infarction caused during reperfusion injury as compared to control (32.2 ± 1.6% vs. 47.4 ± 2.5%, respectively, p<0.01, Figure 5). Additionally, treatment with mdivi-1 also attenuated the exacerbation of the ischaemia/reperfusion injury caused by doxorubicin treatment as compared to doxorubicin alone (45.8 ± 3.8% vs. 69.4 ± 7%, respectively, p<0.01, Figure 5).

### Mdivi-1 protects against doxorubicin-induced depolarisation and hypercontracture of cardiac myocytes

**Figure 6:**
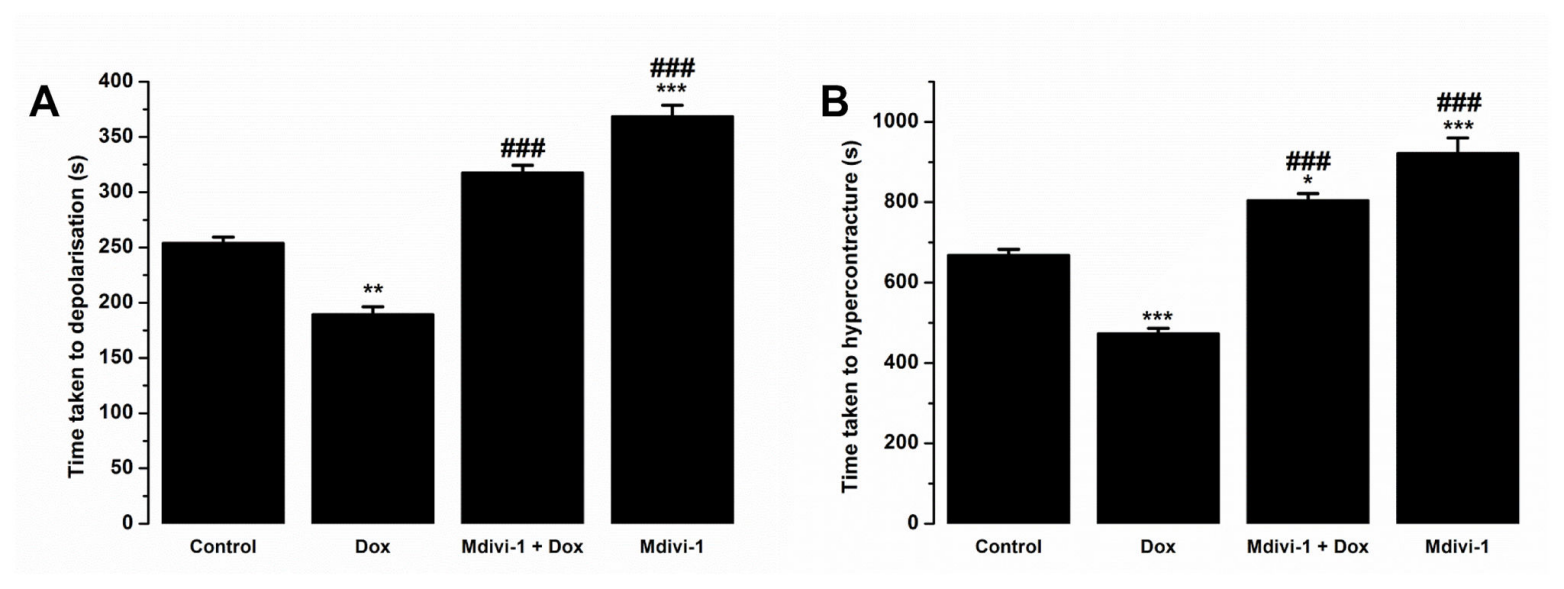
The effects of doxorubicin, Mdivi-1 and the co-treatment of doxorubicin and Mdivi-1 on the time taken to (A) depolarisation and (B) hypercontracture of the rat cardiac myocytes under the conditions of sustained oxidative stress. *p<0.05 vs. control, **p<0.01 vs. Control, ***p<0.001 vs. Control and ###p<0.001 vs. doxorubicin, n=6 (each n comprising of 20±5 cells).

Opening of the mitochondrial permeability transition is found to be involved in doxorubicin induced cardiotoxicity [29,32]. Figure 6a shows the effects of drug treatment on the time taken to the depolarisation of the cardiac myocytes following persistent oxidative stress. Doxorubicin-treated cells depolarised significantly faster (p<0.05) as compared to vehicle-treated cells (189.2 ± 7.1%s vs. 253.6 ± 5.8s, respectively, Figure 6a), while co-treatment of doxorubicin with mdivi-1 significantly attenuated the observed effects of doxorubicin alone (317 ± 7s vs. 189.2 ± 7.1s, p<0.001, respectively, Figure 6a). Doxorubicin treatment caused a significant (p<0.01) reduction in the time taken to hypercontracture of cardiac myocytes as compared to cells treated with vehicle control (472 ± 14.2s vs. 667.7 ± 15.3s; Figure 6b). Treatment with mdivi-1 alone significantly (p<0.001) delayed the time taken to hypercontracture as compared to control (921.4 ± 38.6s vs. 667.7 ± 15.3s, respectively, Figure 6b). Interestingly, the combination of doxorubicin with mdivi-1 reduced (p<0.001) the effects of doxorubicin alone on the time taken to hypercontracture (804.7 ± 16.7 vs. 472 ± 14.2s, respectively, Figure 6b). 

### Effect of mdivi-1 on doxorubicin-induced effects on Erk 1/2 and Akt signalling protein levels

**Figure 7:**
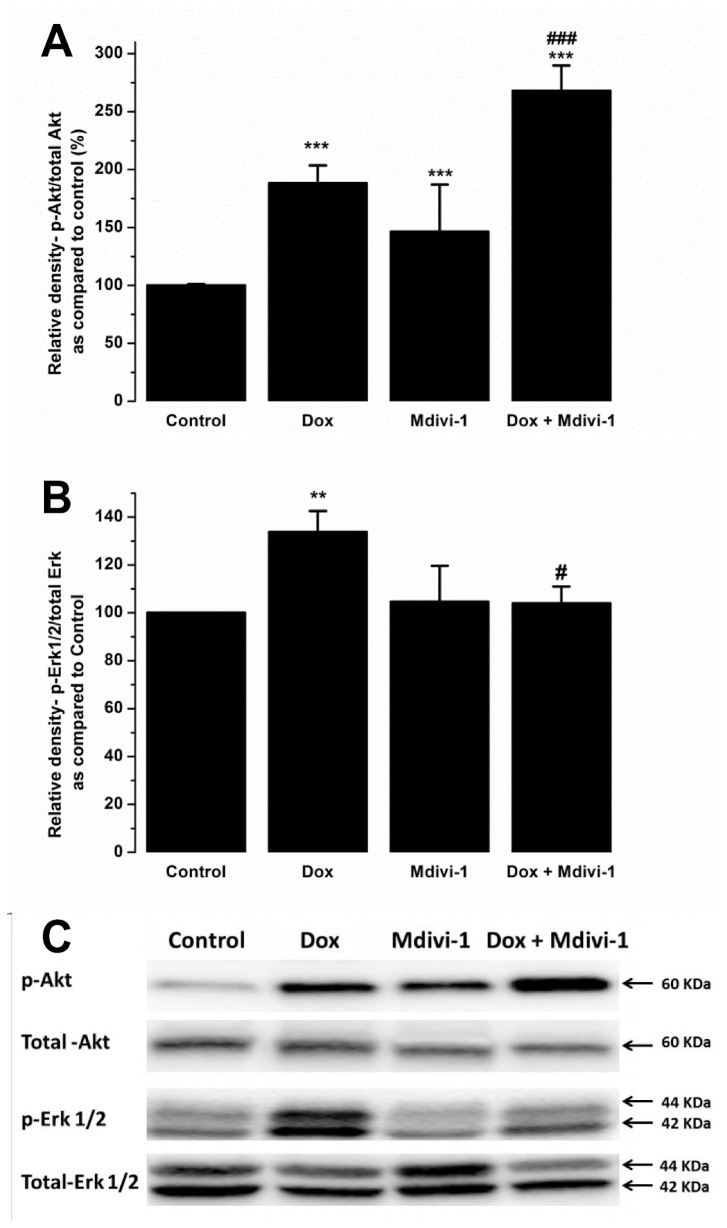
The effects of Dox, Mdivi-1 and co-treatment on the levels of phosphorylated (A) Akt and (B) Erk 1/2. (C) Representative blots showing p-Akt, total Akt, p-Erk 1/2 and total Erk 1/2. **p>0.01 vs. control , ***p<0.001 vs. control, #p<0.01 vs. doxorubicin and ###p<0.001 vs doxorubicin.

Western blot analysis upon treating Langendorff hearts with doxorubicin or mdivi-1 (alone and in combination showed that doxorubicin treatment caused approximately 88% increase in the levels p-Akt as compared to vehicle treated samples. Co-treatment of doxorubicin with mdivi-1 and administration of mdivi-1 alone also caused increase in the p-Akt levels as compared to control (268% and 146%, respectively, Figure 7a). Furthermore, co-treatment of doxorubicin with mdivi-1 also produced significance when compared with doxorubicin alone (268 ± 21% vs 188 ± 7%, respectively, Figure 7a). We also investigated the effects of drug treatment on Erk 1/2 phosphorylation and found that doxorubicin alone caused a significant increase (by 34%) in the levels of Erk 1/2 phosphorylation whereas treatment with mdivi-1 alone did not have any effect on Erk 1/2 levels. Interestingly, co-treatment of doxorubicin with mdivi-1 significantly attenuated the increase in p-Erk 1/2 observed with doxorubicin alone (104 ± 7% vs. 134 ± 8.9%, p<0.05, respectively, Figure 7b).

### Effect of mdivi-1 on doxorubicin-induced effects on phospho-Drp1 and phospho-p53 levels

**Figure 8:**
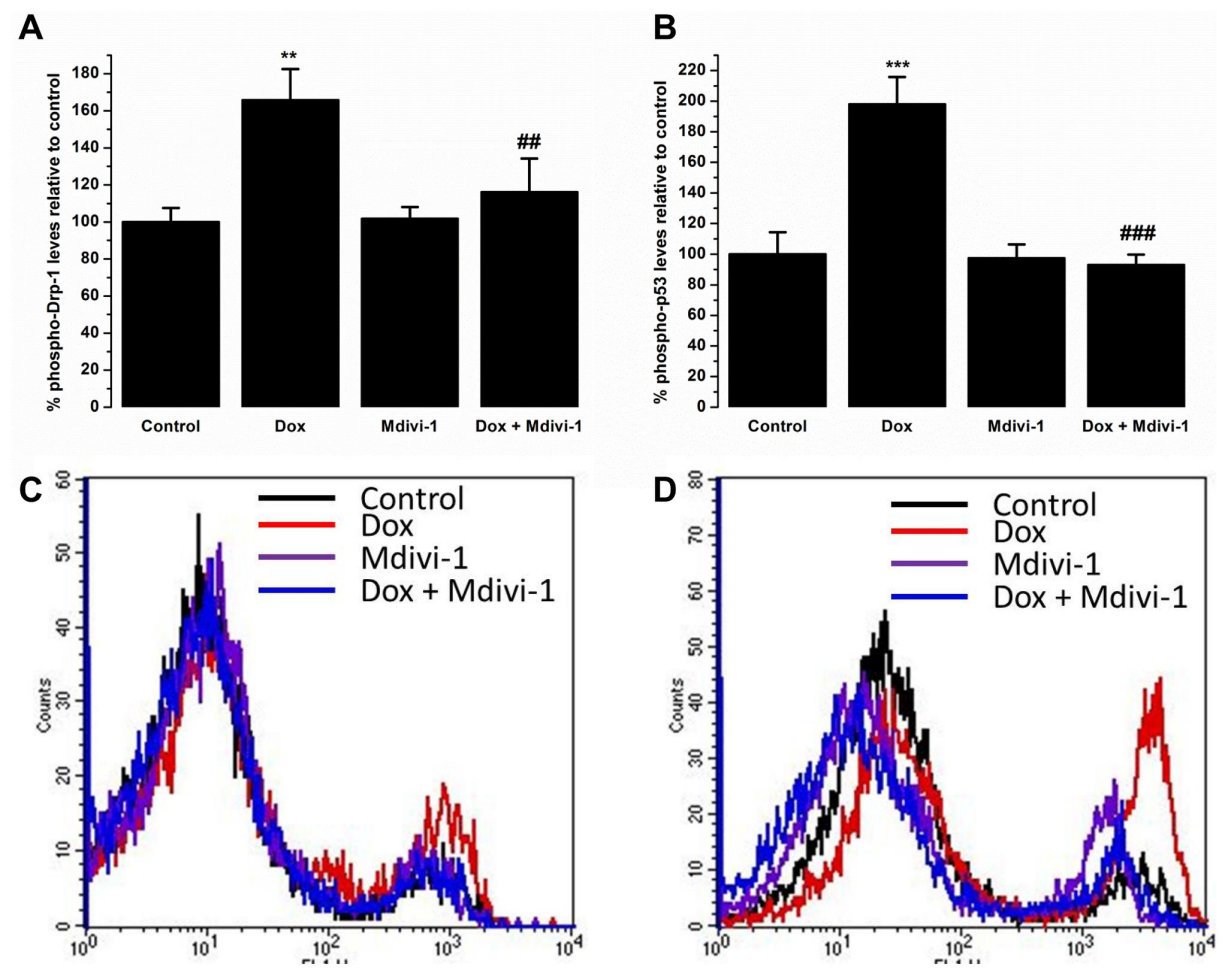
The effects of Dox, Mdivi-1 and co-treatment on the levels of (A) phospho-Drp1 and (B) phospho-p53 with the representative flow cytometry traces of (C) phospho-Drp1 and (D) phospho-p53. **p>0.01 vs. control, ***p>0.01 vs. control, ##p<0.01 vs. doxorubicin and ###p<0.01 vs. doxorubicin.

Flow cytometric analysis of phospho-Drp1 (p-Drp1) levels showed that treatment with doxorubicin (1µM) caused a significant (p<0.01) up regulation in the levels of p-Drp1 when compared to control (165.7 ± 16.8 % vs. 100 ± 7.5 %, respectively, Figure 8a). Interestingly, co-treatment of doxorubicin with mdivi-1 significantly (p<0.01) abrogated the increase in p-p-Drp1 levels caused by doxorubicin alone (116.1 ± 18.1% vs. 165.7 ± 16.8 %, respectively, Figure 8a). Treatment with mdivi-1 alone did not cause a significant change in the levels of p-Drp1 when compared to control (101.7 ± 6.3% vs. 100 ± 7.5%, respectively, Figure 8a). Treatment with doxorubicin also caused a significant (p<0.001) increase in the levels of phopho-p53 (p-p53) when compared to control (198 ± 17.8% vs 100 ± 14.4%, respectively, Figure 8b). Co-treatment of doxorubicin with mdivi-1 prevented (p<0.001) the increase in the p-p53 levels caused by doxorubicin alone (92.9 ± 6.9% vs 198 ± 17.8%, respectively, Figure 8b). Treatment with mdivi-1 alone did not have an effect on the levels of p-p53 when compared to control (97.3 ± 9.1% vs 100 ± 7.5%, respectively, Figure 8b).

### Co-incubation of mdivi-1 with doxorubicin does not alter the cytotoxicity of doxorubicin against leukaemic HL60 cells

**Figure 9:**
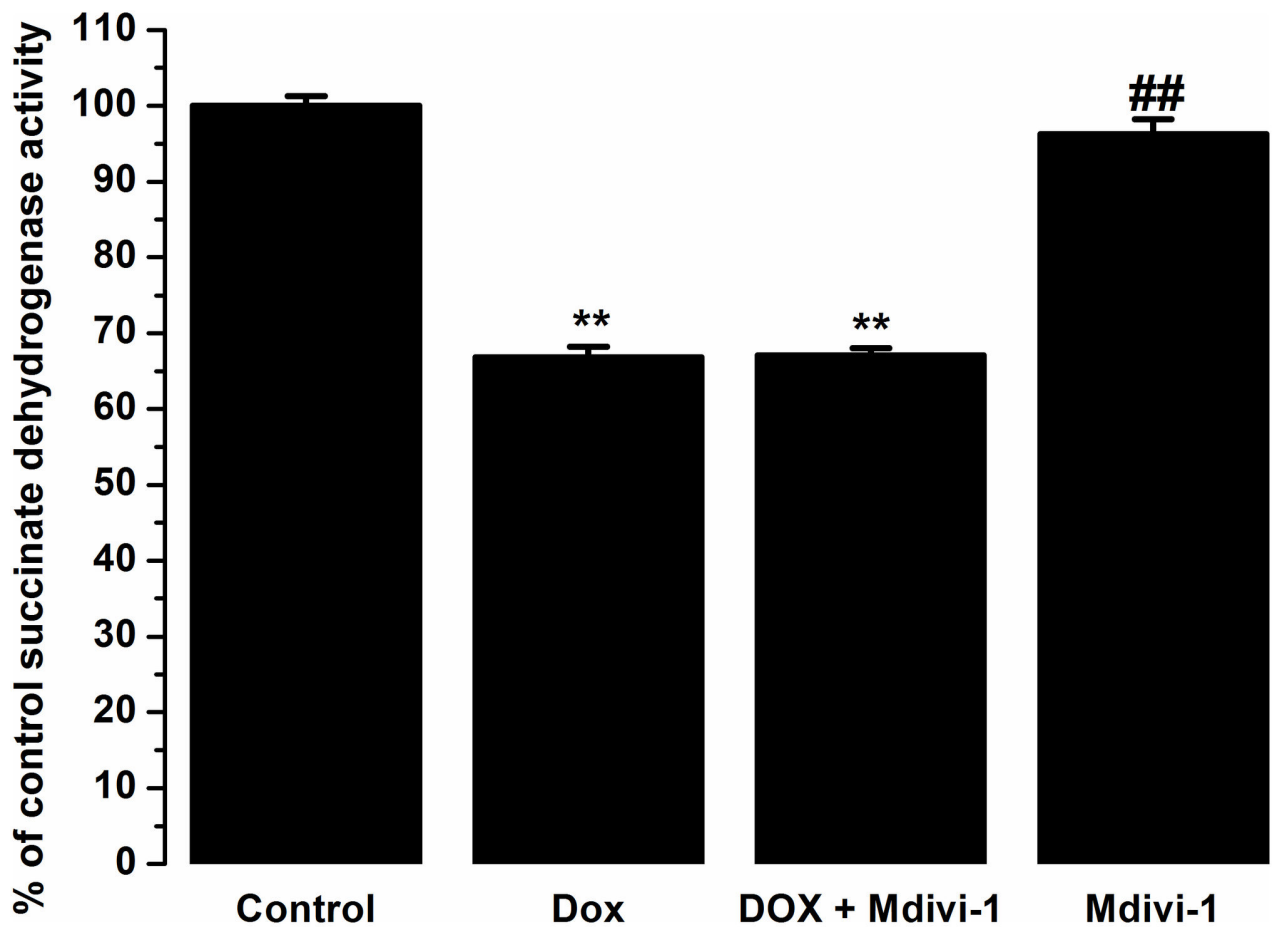
The effects of Dox, Mdivi-1 and co-treatment on the MTT reductase activity as compared to control on HL60 cancer cells. **p>0.01 vs. control, ##p<0.01 vs. doxorubicin.

The colorimetric MTT assay demonstrated as expected that doxorubicin reduced the viability of HL60 cells by 33% as compared to the non-treated controls (p<0.01). Co-administration of doxorubicin with mdivi-1 did not alter the anti-cancer activities of doxorubicin alone. Similarly, mdivi-1 alone did not have any effects on the cell viability of the cells as compared to control (Figure 9).

## Discussion

Doxorubicin treatment is known to cause cardiovascular toxicity due to the generation of reactive oxygen species [5] and calcium overload [33]. Previous research has demonstrated that doxorubicin induced toxicity affects mitochondrial bioenergetics and causes mitochondrial fragmentation [17]. We demonstrate that doxorubicin induced dysfunction on the haemodynamic parameters of the hearts are reversed by mdivi-1, a relatively specific inhibitor of mitochondrial division.

Doxorubicin induced effects of cardiac function has been reported in *in vivo* and *in vitro* studies. Doxorubicin has previously been found to reduce both left ventricular developed pressure and heart rate, also shown in this study [15,34,35]. Interestingly, the presented data show that doxorubicin treatment in the naïve hearts caused a drop in the heart rate readings as opposed to its effects in conditions of ischaemia and reperfusion injury where no significant decrease in the heart rate values were recorded. One possible explanation for this effect could be the level of oxygen, previously published work has indicated that doxorubicin-induced decrease in the heart rate was more prominent when the heart were perfused with 95% oxygen as compared to 20% oxygen [34]. We also show that co-treatment with mdivi-1 abrogated the detrimental effects of doxorubicin on left ventricular developed pressure. Interestingly, treatment with mdivi-1 was shown to ameliorate left ventricular dysfunction caused by pressure overload heart failure as assessed by left ventricular chamber diameter and fractional shortening [27]. Mitochondrial fragmentation is proposed to be a major player in exacerbation of heart failure, inhibition of fragmentation is therefore thought to confer cardioprotection [27]. Recent research has indicated that mitochondrial dynamics play a crucial role in cell physiology and growing evidence suggests that a balance between mitochondrial fission and fusion plays a vital role in pathological conditions [23,36,37]. Studies have also shown that mitochondrial oxidative stress, which is also induced by doxorubicin treatment [1,5], leads to fragmentation of the mitochondria, which were attenuated with reactive oxygen species scavengers [38]. Mitochondrial fragmentation has been found to mediate cellular function and apoptosis [37,39]. Mdivi-1 has been suggested to have therapeutic potential for a variety of diseases such as stroke, myocardial infarction and neurodegenerative disorders [23,26,40]. In the current study, flow cytometric analyses of p-Drp1 levels show a significant up regulation of p-Drp1 levels following treatment with doxorubicin, which was prevented when doxorubicin was co-administered with mdivi-1. Elevated levels of mitochondrial fission proteins have been reported in response to ceramide and doxorubicin induced toxicity [17]. It has been demonstrated that mdivi-1 inhibits GTPase activity by blocking self-assembly of Drp1, preventing mitochondrial fission [40]. It has been postulated that doxorubicin induced cardiotoxicity involves fragmentation of the mitochondria. A recent study has shown that doxorubicin treatment leads to an increase in GTPases that are found to govern mitochondrial fission and fusion [41]. Imbalance in mitochondrial dynamics has been found to play a critical role in the pathophysiology of the failing heart [42]. Therefore, modulation of mitochondrial fission and fusion machinery could therefore compensate for the detrimental effects of doxorubicin on mitochondrial bioenergetics [17,41,43]. 

In the current study we demonstrate for the first time the effects of mitochondrial division inhibition (mdivi-1) on doxorubicin induced cardiotoxicity in naïve and in the conditions of ischaemia and reperfusion injury. We demonstrate that co-administration of mdivi-1 with doxorubicin significantly reduced doxorubicin-induced myocardial dysfunction and infarction in both conditions. Whether doxorubicin-induced effect on coronary flow was a direct effect on coronary endothelial cells or secondary to the injury of cardiac myocytes was not further investigated. It is critical to investigate therapeutic potential of anti-cancer compounds in stressed conditions such as ischaemia or reperfusion injury, as cancer and cardiovascular diseases are likely to co-exist in patients. It has also been shown that anti-cancer drugs can lead to or exacerbate the risk of cardiomyopathy when baseline heart diseases are taken into account [12]. An analysis of 5-year cancer survivors has found that a huge number of cancer patients on anthracyclines die from cardiac related causes [44]. They also showed that cancer treatments in childhood cancers increased the risk of congestive heart failure 15 fold compared to the non-treated [44,45]. Furthermore, we have recently shown that doxorubicin administration at the time of reperfusion exacerbates ischaemia and reperfusion injury, which was abrogated with co-treated with mPTP blocker cyclosporin A [29]. Hence, cancer therapeutics may prove to exacerbate underlying cardiovascular diseases, as we have shown in this study, and it is important to assess potential adjunct therapies in pathological conditions as well as in naïve conditions.

Recently, experimental evidence has highlighted multiple beneficial effects of mdivi-1 treatment by specific and non-specific actions by reducing ischaemia reperfusion injury [26] and pressure induced heart failure [27]. Mdivi-1 treatment is also found to directly inhibit the rapidly activating delayed-rectifier K^+^ currents in HL-1 cells in a concentration dependent manner [46]. The protective effects of mdivi-1 in doxorubicin-induced cardiotoxic effects observed in this study could be a direct effect on mitochondrial fragmentation, which is found to occur simultaneously with the release of cytochrome c [39]. It has also been noted that Drp1 inhibition interferes with the apoptotic process, without completely inhibiting cell death [47]. Direct effects of mdivi-1 on cardiac myocyte mitochondria were shown previously in myocardial ischaemia reperfusion injury [26]. Our results show that doxorubicin treatment caused rapid depolarisation and hypercontraction of cardiac myocytes as compared to non-treatment following persistent oxidative stress, a similar effect of oxidative stress on mitochondrial energetics and permeability transition has previously been reported [17,41,48,49]. We also show that mdivi-1 caused a delay in depolarisation and hypercontraction, confirming previous reports on the protective effects of mdivi-1 on ROS induced mPTP opening [26]. Interestingly, co-treatment of doxorubicin with mdivi-1 protected against doxorubicin-induced effects on depolarisation and hypercontraction. These findings further confirm the involvement of mitochondrial fission in doxorubicin-induced cardiotoxicity and suggest that pharmacological modulating mitochondrial fission may have cardioprotective effects, which could also directly affect the mPTP [48,49]. 

Western blot analysis were carried out to investigate the effects of drug-treatment on the levels of survival kinases Akt and Erk 1/2 as well as to investigate whether the protective mdivi-1 against the damaging effects of doxorubicin involve these pathways. We observed that treatment with doxorubicin increased the levels of the survival proteins, p-Erk 1/2 and p-Akt. Doxorubicin-induced increase in the levels of Akt could either be a direct effect of doxorubicin on the cardiac myocytes in the heart or an indirect effect of the heart that is initiated in order to protect against the damaging effects of doxorubicin. Previous studies have indicated this effect of doxorubicin treatment on survival proteins and it has been suggested that it may serve as an endogenous protective effect of the heart to protect against the toxic effects of doxorubicin [50–52]. Downstream effectors of Akt and Erk converge to the mitochondria and initiate a protective response [53]. There is additional evidence that coronary delivery of constitutively active form of Akt1 gene protects the heart against doxorubicin-induced chronic heart failure by improving cardiac performance [54]. We postulate that the increase in the pro-survival proteins observed in this study serves as an innate mechanism of the heart to protect against the damaging effects of doxorubicin. We also show increased p-Akt levels when treated with mdivi-1 alone and a further increase when treated with the combination of mdivi-1 and doxorubicin. A link between Akt pathway and the mitochondrial fusion and fission mechanism has been suggested previously [23]. It is believed that increase in Akt phosphorylation promotes mitochondrial fusion, which is considered to lead to its cardioprotective effects. It has also been reported that compounds which offer cardio-protection such as insulin or anti-oxidants prevent ischaemia induced fragmentation and produces elongated mitochondria [55]. It has also been speculated that the cytokine erythropoietin induces mitochondrial fusion by activating Akt [23]. However, a downstream effector of Akt, protein kinase G (PKG), has been reported to phosphorylate and inhibit the pro-fission activity of Drp1. A recent study reported increase in the levels insulin stimulated Akt phosphorylation when also treated with mdivi-1 [56]. Further investigations are needed to establish whether mdivi-1 treatment causes a direct effect on Akt phosphorylation. We speculate that the huge increase observed in Akt phosphorylation when co-treated with doxorubicin and mdivi-1, is due to the dual effect of doxorubicin (innate response of the heart) and a direct effect of mdivi-1 on Akt phosphorylation causing a further increase. Previously, we reported a significant increase in the levels of p-Akt following doxorubicin-treatment in conditions of ischaemia and reperfusion injury, which was partially blocked when co-administered with cyclosporin A as well as providing protection against the toxic effects of doxorubicin [29]. Further studies are required to investigate the exact role of doxorubicin-induced toxicity and the protection there from with adjunct therapy on Akt phosphorylation. Studies have also reported a link between ROS generation, mitochondrial morphology and Erk 1/2 signalling in the regulation of insulin signalling pathway. Obesity induced ROS appeared to increase the levels of Erk 1/2 phosphorylation, which were reversed when treated with mdivi-1 [56]. A similar effect is also seen in our data showing that co-treatment with mdivi-1 reverses doxorubicin induced increase in Erk 1/2 levels. Furthermore, it has been reported that doxorubicin-induced involves Erk/p53 transduction pathway [57]. Treatment of H9c2 and cardiac myocytes with doxorubicin caused an increase in the levels of p53 which were preceded by activation and nuclear translocation of Erk 1/2 [57]. They also showed that inhibition of Erk 1/2 with U-0126 prevented activation and nuclear translocation of both Erk 1/2 and p53 whilst inhibition of p53 with pifithrin-α only prevented doxorubicin-induced changes in p53 [57]. In the current study we show an increase in the levels of p53 and Erk 1/2 following treatment with doxorubicin. Interestingly, co-administration of doxorubicin with mdivi-1, which prevented detrimental effects of doxorubicin in the Langendorff and oxidative stress model and reduced the levels of p-Drp1, also prevented the increase in the levels of both p53 and Erk 1/2. Further studies are required to identify the specific role of Erk 1/2 and Akt activation using their specific inhibitors and the intracellular signalling pathway associate with the protective effect mdivi-1.

Furthermore, we show that co-treatment with mdivi-1 does not interfere with the anti-cancer properties of doxorubicin as assessed by MTT assay using HL60 cells (Figure 9). It is imperative to assess the effects of adjunct therapies, aiming to reduce the cardiotoxic effect, on the anti-tumour effects. Many cardioprotective strategies fail to demonstrate beneficial effects in clinical or *in vivo* settings as they interfere or reduce with the anti-cancer effects and thereby reduce the clinical utility [14].

Collectively, our data show that co-treatment with the mitochondrial fission inhibitor mdivi-1 can ameliorate the cardiotoxic effects of doxorubicin without affecting its anti-cancer properties. These finding warrant further investigations in the relevant animal models of cancer. 
